# Overweight and Obesity in Dogs and Cats: An Exploration of Animal Welfare and Behaviour Impacts, and Recommendations for Management in Veterinary Primary Care

**DOI:** 10.3390/ani16081204

**Published:** 2026-04-15

**Authors:** Rimini Quinn, Anne Quain

**Affiliations:** Sydney School of Veterinary Science, Faculty of Science, University of Sydney, Camperdown, NSW 2006, Australia

**Keywords:** overweight, obesity, body condition score, animal welfare, animal behaviour, primary care, veterinary practice, dogs, cats

## Abstract

Overweight and obesity are prevalent among companion dogs and cats in the Western world. Affected animals are at risk of comorbidities and reduced longevity. This narrative review found that veterinary literature generally characterises overweight and obesity as nutritional disorders that are primarily addressed by reducing caloric intake. However, veterinary management of overweight and obesity has limited success outside research settings. The Five Domains model for animal welfare assessment is applied to explore impacts of overweight and obesity and their management in dogs and cats by focusing on nutrition, health, physical environment, behavioural interactions and mental state. A second focus is on the practical strategies for addressing non-diet-related barriers and client communication through the provision of management recommendations. This novel and integrative approach aims to inform veterinarians and improve the success of weight management protocols.

## Simple Summary

Overweight and obesity are common conditions seen in dogs and cats in veterinary general practice that may negatively impact animal welfare. Obesity in particular is associated with reduced quality and quantity of life in dogs and cats. Overweight and obesity stem from a chronic positive energy balance, typically viewed as a nutritional disorder. We argue that this narrow conception omits consideration of the welfare and behaviour implications of both overweight and obesity and interventions to successfully address these. This in turn may compromise communication with clients, leading to failure of overweight and obesity management. We provide a narrative review of recent veterinary literature on overweight and obesity, with an emphasis on welfare, behaviour and veterinary management. We then provide recommendations that may inform management of overweight and obesity by veterinary general practitioners, and future research into overweight and obesity in companion animals.

## 1. Introduction

Overweight and obesity are among the most common diagnoses in veterinary primary care, accounting for 6.6–11.6% of clinical diagnoses [1,2,3,4,5]. This is likely, at least in part, because the diagnosis can be made based on a history and physical examination [2]. In an investigation of common clinical presentations in primary care consultations in the UK, overweight and obesity were the most common problems identified clinically, and the most common non-presenting findings, but—like dental disease and behavioural concerns—were not identified as common presenting problems [1]. In other words, caregivers typically did not present animals because they were overweight or obese. Veterinarians had to actively identify these issues.

Management of overweight and obesity requires the promotion of a negative energy balance to minimise excess adipose tissue [6]. This is achieved through reduced caloric intake and, where possible, increased caloric output. A secondary aim is to minimise loss of lean muscle mass, which increases basal metabolic rate, by ensuring an appropriate diet and providing adequate exercise [7]. Overweight and obesity are thus considered primarily disorders of nutrition. In our experience, this limited conception overlooks the welfare and behavioural drivers and impacts of overweight and obesity in dogs and cats, which can negatively influence management outcomes. In this review, we explore the welfare and behavioural impacts of overweight and obesity and their management in dogs and cats. We provide general recommendations for veterinary primary care teams to ensure that welfare and behaviour considerations are accounted for in the management of overweight and obesity.

## 2. Materials and Methods

A narrative methodology was selected, aligned with subjectivist and interpretivist paradigms, as the most appropriate method of summarising key information and practice-relevant insights from a heterogeneous body of literature on the intertwined topics of prevalence, diagnosis, consequences and management of overweight and obesity in dogs, in addition to providing critical interpretation [8,9,10]. Our aim was to produce an overview of overweight and obesity in dogs and cats, with an emphasis on animal welfare and animal behaviour, to inform veterinary team members who manage these conditions. Narrative reviews are best suited to include a broad variety of studies and provide an overall summary, critique and interpretation, and may not include all literature on a topic [8].

As narrative reviews are enhanced when researchers are explicit about how their perspectives and experiences informed problem identification, interpretation and analysis [8], we provide positionality statements in Section 2.1.

While not a systematic review, we used transparent inclusion and exclusion criteria with explicit thematic synthesis. Inclusion criteria were that articles were (1) published in the English language; (2) available as full-text articles, reports or web pages; and (3) substantially focused on overweight or obesity in dogs and cats. Exclusion criteria were (1) not available in the English language; (2) not available as full-text articles, reports or open-access web pages; (3) focused on medical causes of obesity; and (4) focused on non-dog and cat species. Literature searches were conducted in April 2025 and again in December 2025, using Web of Science and PubMed databases. Additional searches were performed using Google Scholar to ensure as many relevant articles as possible were captured. Citations in articles retrieved were screened for other relevant articles. Additional ad hoc searches were undertaken during the development of this manuscript, including editing. Key search terms included overweight, obesity, body condition score, dog, cat, veterinarian, welfare and behaviour. Titles and abstracts were screened for relevance. Grey literature was included to expand the material available for review. Additional search terms included weight stigma, weight loss, feeding and foraging behaviour, and client communication. We sought to include literature that made explicit mention of or had clear implications for animal welfare in relation to overweight and obesity, and practical management of overweight and obesity. Because we sought to include a wide range of study designs, no methodology quality assessment or classification of evidence according to a hierarchy was undertaken. We did not set a date range; however, to ensure currency, we preferentially cited the most recent literature (published in the previous 5–10 years) where available.

**Figure 1:**
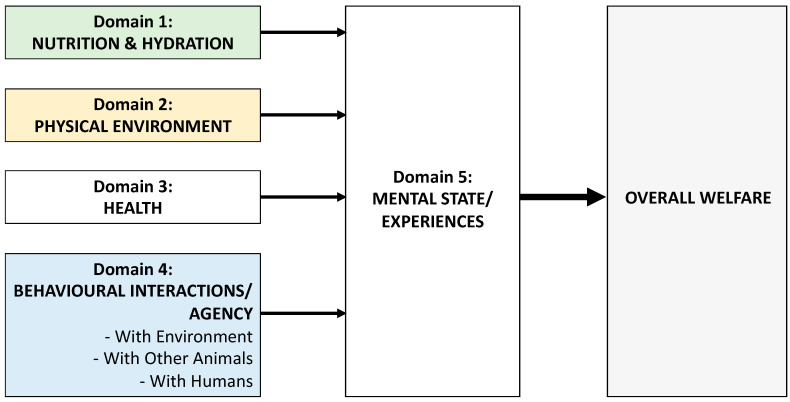
The Five Domains Model for Assessing Animal Welfare, reproduced with permission from Littlewood, K. (2025) Case 5.4 in *The Veterinary General Practice Case Book: Companion Animal Clinics*, edited by Andrew Gardiner and Anne Quain, p. 256 [12].

We employed the Five Domains model [11] of animal welfare as a framework to understand reported impacts of overweight and obesity on animal welfare, including behavioural interactions and agency. The Five Domains model considers welfare as what an animal experiences overall (their mental state/experiences, Domain 5). Domains 1–4 (nutrition and hydration, physical environment, health and behavioural interactions/agency) all lead to affective states reflected in Domain 5 (e.g., not enough food leads to hunger, an inappropriate physical environment might lead to thermal discomfort, poor health may cause pain, malaise and discomfort, lack of agency may lead to frustration and boredom), all of which contribute to an animal’s overall welfare [11] (Figure 1).

Because we have no direct insight into affective or mental states (Domain 5), the objective information about an animal’s welfare is derived from assessing Domains 1–4, identifying areas of concern, addressing these and re-evaluating the animal’s welfare [11]. That said, evidence from affective neuroscience supports specific positive and negative affective states associated with inputs from Domains 1–4 [13]. This provides insights into affective states that animals experience [11], which we have included where relevant. For the purposes of this discussion, the terms “owner”, “caregiver” and “client” are used interchangeably, as all are used in the literature.

### 2.1. Positionality Statements

The first author (RQ) is a veterinarian who worked in small animal practice for 15 years before gaining Membership in Veterinary Behaviour with the Australian and New Zealand College of Veterinary Scientists (ANZCVS), transitioning to work as a behaviour veterinarian. She has worked in veterinary behaviour practice for 10 years, treating dogs and cats with behaviour problems. She is concurrently completing her PhD in Canine Behaviour and Welfare, focused particularly on chewing behaviour. Her relevant interests include how behaviour and overweight and obesity influence each other, particularly through the ethology of appetitive and consummatory behaviour, psychology (especially around learning theory), and the human–animal bond. She is enthusiastically assisted in her current chew studies by her rescue dog (and chief chew tester), Stryder, and her two cats.

The second author (AQ) is an Australian-trained veterinary general practitioner. As a veterinary student, she collaborated on a project exploring the incidence of overweight and obesity in dogs and cats in Australia. She is a Diplomate of the European College of Animal Welfare and Behaviour Medicine (ECAWBM) in Animal Welfare Science, Ethics and Law (AWSEL) and a Member of ANZCVS Animal Welfare chapter. She teaches veterinary ethics, professional practice and animal welfare in an Australian veterinary school. Professionally, she encounters overweight and obese animals in primary care settings, and it is her subjective impression that despite advances in knowledge regarding animal health and nutrition and the widespread availability of commercial weight loss diets for dogs and cats, weight management strategies focused on nutrition and exercise generally fail. Personally, her family includes both a 14-year-old Domestic Medium Hair cat on a prescription diet for urolithiasis and a two-year-old Labrador retriever, both of whom have high food motivation.

## 3. Review

### 3.1. Definitions of Overweight and Obesity in Dogs and Cats

Overweight and obesity occur on a continuum of excess weight, with obesity at the far end of the continuum. The Global Pet Obesity Initiative defines 5 on a nine-point system as the ideal body condition scoring (BCS) [14]. Each category between 5 and 9 represents approximately 10% excess weight. According to this system, obesity in dogs and cats is 30% above ideal body weight, equivalent to 8/9 [14], with a BCS of 9, equivalent to being 40% above ideal weight [15]. According to this system, overweight (BCS > 5 and <8) is defined as 1 to 29% above ideal weight. This degree of precision is unlikely to be met in practice, where overweight is pragmatically defined using the 9-point body condition scoring system (equivalent to 10 and 20% above ideal weight). The term “Class I obesity” refers to animals 30–40% over ideal weight, while “Class II obesity” refers to animals > 40% over ideal weight [15]. For the purposes of this discussion, we use the Global Pet Obesity Initiative definitions except where cited studies refer to Class I and Class II obesity, in which case we use these terms. The so-called “gold standard” method of diagnosis is dual-energy X-ray absorptiometry (DEXA), but access is limited to veterinary research and specialist facilities [15]. Body condition scoring remains the most practical means of diagnosis of obesity in primary care settings.

### 3.2. Prevalence of Overweight and Obesity in Dogs and Cats

Obesity has been described as “the most common age-related disease in cats” [16] and “one of the most significant and preventable contributors to age-related morbidity and reduced lifespan in dogs and cats” [5]. In Australia, where the authors practise, the prevalence of overweight and obesity was 33.5% and 7.6%, respectively, in dogs [17] and 26.2% and 6.6%, respectively, in cats [18]. A recent, non-peer-reviewed report found that the percentage of Australian cats classified as overweight or obese was 17% in the first year of life, 37% from the second year and 53% in cats 7–8 years of age [19].

A French study based on veterinarians’ body condition scoring found that 47.5% of cats were overweight and 2.9% were obese [20]. A retrospective study of electronic patient records (EPRs) reported a one-year prevalence of overweight or obesity of 7.1% in the United Kingdom (UK) [21]. A similar study examining EPRs from veterinary clinics in New Zealand reported that 26.1% and 2.3% of dogs were overweight and obese, respectively, while 21.9% and 2.6% of cats were overweight and obese, respectively [22]. A cross-sectional survey of dog owners (*n* = 3185) in 10 European countries (Croatia, Denmark, Italy, Lithuania, Poland, Portugal, Romania, Serbia, Spain and Sweden) found owner-reported rates of overweight and obesity based on body condition scoring in dogs to range from 6.0% to 31.3% [23]. An epidemiological study of dogs visiting 14 animal hospitals across 7 districts in Beijing reported an obesity rate of 44.4% [24]. In a study of client-owned adult cats in Zealand, Denmark, 35% were classified as “heavy/obese” (BCS 7–9/9), as determined by veterinary investigators during home visits [25].

A retrospective observational study of over 4.9 million dogs and 1.3 million cats visiting one chain of veterinary hospitals in the US between 2020 and 2023 reported the prevalence of overweight and obesity, respectively, according to life stages in dogs, as 0.9% and 0.0% (early growth), 9.5% and 0.3% (late growth), 24.4% and 1.9% (young adult), 44.5% and 8.4% (adult), 50.1% and 12.6% (mature) and 46.4% and 11.3% (senior) [26]. In cats, the prevalence of overweight and obesity, respectively, according to life stages, was 0.8% and <0.0% (early growth), 10.7% and 0.4% (late growth), 36.2% and 3.6% (young adult), 47.2% and 13.9% (adult), 44.8% and 21.7% (mature) and 32.0% and 12.6% (senior). While the prevalence of overweight and obesity was low in puppies and kittens, those observed to be overweight or obese were almost twice as likely to be overweight or obese in adulthood. In the Cat Prospective Ageing and Welfare Study, a longitudinal study of client-owned pet cats, feline body weight and body condition score increased during middle age up to nine years, before declining after the age of 10 [27]. The frequency of being either overweight or obese was stable until 12 years (47.0%) and 10 years (14.5%), after which this declined to 33.2% and 0.4%, respectively, by the age of 16. In this study, muscle condition score declined from middle age. Male cats were heavier than females and 2.8 times more likely to be overweight or obese.

Overall, overweight and obesity are more prevalent in adult animals and tend to peak in mature life stages before declining in the senior stage. Heterogeneity in overweight and obesity prevalence between studies and countries may reflect different methodologies for assessing overweight and obesity or collecting data, or variation in genetic or environmental risk factors. Nonetheless, a global survey of practising veterinarians (*n* = 1167) carried out by the Animal Welfare Working Group of the World Small Animal Veterinary Association found that obesity was considered the most important animal welfare issue in most geographic regions [28].

### 3.3. Animal and Client Risk Factors for Overweight and Obesity in Dogs and Cats

Overweight and obesity typically stem from a chronic positive energy balance [6], with consumption of high-calorie food and insufficient physical activity increasing the risk. Chronic positive energy balance in turn leads to fat accumulation and alteration in body condition [7]. As Wallis and Raffan state, “Consequently, obesity has commonly been considered a ramification of poor self-control in people or of inept management by animal owners. However, considerable evidence now shows obesity is better regarded as a disease of disordered energy homeostasis in which a multitude of genetic and environmental factors can contribute to increasing body fat” [29].

Animal-related risk factors include food drive, sex and neutering status, increased age, orthopaedic conditions, reduced activity (including confinement in cats), polyphagia (e.g., due to endocrinopathies or medications like glucocorticoids) and breed [6,20,24,30,31,32]. A retrospective review of body weight, BCS and patient demographics from electronic medical records of cats in the UK found that cats neutered prepubertally (4 months of age or less) showed no difference in body weight or BCS from those neutered at 5–6 months, but those neutered at 7–12 months had a lower risk of body weight and BCS gain overall [33]. Factors associated with overweight and obesity in cats included being middle-aged, being male, being neutered, being mixed breed, having low physical activity and begging for food [20,25,34,35]. Conversely, animals with higher activity are less likely to be overweight or obese.

The Dog Obesity Risk and Appetite (DORA) questionnaire revealed that high food motivation was associated with obesity in dogs [36,37]. Food motivation had a similar magnitude of effect to both exercise and owners giving their dogs human food—suggesting that dogs’ food motivation is as important a risk factor for obesity as owner factors [36]. Food motivation score was higher in American Kennel Club sporting and hound breed groups (including Labrador retrievers and Golden retrievers), and the UK gun dog group (including Labrador retrievers) compared with mixed-breed dogs and non-sporting breed groups [36,37]. Some breeds are at increased risk of obesity. For example, deletion of the pro-opiomelanocortin (POMC) gene in Labrador and flat-coated retrievers was associated with increased body weight, adiposity and food motivation [29,38]. The polygenic risk score is more common in assistance dogs than pet Labradors. This is possibly because high food motivation improves dog responsiveness to food used as positive reinforcement during training [39].

Client-related risk factors for animal overweight and obesity include reduced physical activity or mobility constraints, age, gender, an inactive lifestyle, smoking, not considering obesity a disease, being strongly attached to their companion animal, providing dry food as the primary diet, *ad-lib* feeding, confining animals for the majority of the time indoors, and underestimating BCS [23,25,40]. In a study of French cats, those who lived with a child and had a high owner-rated activity score were more likely to have an ideal body condition than be overweight or obese [20]. The authors of this study argue that increased sedentary lifestyles in cats (with 41% of French cats lacking outdoor access in 2020–2022 compared to 29% in 2006) aligns with increased incidence of overweight and obesity [20]. In a study of Danish cats, indoor confinement increased the risk of obesity in cats at an early age, while the risk of obesity in cats with outdoor access was low and slowly increased, peaking at the age of 7 [25].

Low DORA scores on owner factors (including less rigorous control of diet and exercise) were associated with obesity in dogs [36]. Being fed table scraps and snacks was linked with canine overweight and obesity [31,41]. Owners of highly food-motivated dogs tended to exert more control on their dogs’ food intake than owners of less food-motivated dogs. Dogs fed once a day were 1.4 times more likely to be classified by their owners as being obese than those fed more than once per day, and those living in single-dog households were 1.6 times more likely to be classified by their owners as being obese than those in multi-dog households [31]. After controlling for age, sex and weight, dogs in multi-dog households had 3.5% higher food motivation scores than dogs living in single-dog households [37]. Increased weekly exercise was associated with decreased owner-reported obesity in dogs [31]. A survey of Australian cat owners found that those with a positive attitude regarding overweight and obesity were more likely to live with an overweight or obese cat [34].

In a study of 198 human–dog pairs in Spain’s Canary Islands, dogs belonging to overweight owners were over three times as likely to be obese [42]. Similarly, a study of 38 human–dog pairs in the USA found a correlation between owner body mass index and dog body condition score (BCS), supporting a possible association between overweight status in dogs and their owners [43]. The reasons for this correlation require further exploration, but one explanation is that companion animals and humans are exposed to the same “obesogenic” environments [42]. Obesogenic factors, including food marketing practices, institutionally driven reductions in physical activity, increased atmospheric carbon dioxide, increased ambient temperature and time spent in thermoneutral zones, environmental endocrine disruptors and economic disparity and insecurity, may increase the risk of obesity in humans [44] as well as animals. In a prospective study of physical activity, weight status and neighbourhood characteristics of dog walkers across 32 neighbourhoods in the US cities of Seattle and Baltimore, dog owners who walked their dogs were more likely to live in highly walkable neighbourhoods (for example, those with footpaths and other pedestrian facilities) than dog owners who did not walk their dogs [45]. This suggests that owners and dogs share exposure to obesogenic environments. In short-term, paired human-centred and dog-centred weight loss trials, active weight loss in either the owner or dog was associated with passive weight loss in the other, suggesting an effect of the human–animal relationship [46].

An additional risk factor for overweight and obesity may be the widespread availability of nutritionally complete, highly palatable pet food. Dogs and cats readily accept palatable foods. Palatability has been identified as a key driver of the repurchase of particular foods by caregivers, and consequently the success or failure of products on the market [47,48]. Commercial pet food is largely available in dry, semi-moist and moist (wet) varieties with increasing moisture content. Dry food (kibble, biscuits) is extremely popular due to its convenience, longer shelf-life and lower cost compared to semi-moist and moist food. However, it is also typically the more calorically dense. The widespread availability of highly palatable, calorically dense food increases the risk of an energy surplus.

### 3.4. Outcomes of Veterinary Management of Overweight and Obesity

While there are few studies evaluating the efficacy of veterinary management of obesity and overweight in client-owned animals, those that exist suggest low overall success, often with subsequent weight gain. Review of records of dogs referred to a specialist veterinary nutrition team at a US-based teaching hospital for weight loss found that the mean weekly weight loss rate was unsatisfactory in 64% of cases, and 45% of owners did not follow dietary or physical recommendations for their dogs [49]. A retrospective review of overweight and obese dogs seen in four primary care hospitals in the US reported that average weight loss rates were low, and—during treatment for overweight and obesity—the majority of dogs (56.5%) gained weight [50]. In a single-blind, randomised controlled trial (*n* = 106 owners of overweight dogs) where owners in the intervention group were prompted to form “if-then” plans, dogs in the intervention group lost the same percentage of body weight each week as those in the control group [51]. However, around half of the participants dropped out of the study.

In a prospective parallel unmasked block-randomised controlled trial comparing dietary restriction alone (*n* = 9 cats) with an intervention combining dietary restriction, digital scales, smart feeders, activity monitors and treat cameras (*n* = 6 cats), the average weekly weight loss was higher in the technology group than in the dietary restriction group [52]. Four out of nine cats in the dietary restriction group gained weight during the study. In a study of client-owned cats (*n* = 62) referred for weight management, 55% did not reach their target weight, with the most obese cats least likely to do so [53]. In a large, 12-week international multi-centre cohort study of weight loss in overweight client-owned cats (*n* = 730 cats from 188 veterinary practices in 22 countries), those who completed the program lost a mean of 10.6% (+/−6.3%) body weight [54]. Interestingly, cats in North America, especially the USA, lost less weight on average than those from other regions. However, there was a high dropout rate, with 41% failing to complete the trial. Similarly, in a large, 12-week, international multi-centre cohort study of weight loss in overweight client-owned dogs (*n* = 926 dogs from 340 practices in 27 countries), those who completed the program lost a mean of 11.4% (+/−5.84%), with notable differences between dogs of different sex, neuter status and geographic location [55]. In this study, 37% of dogs failed to complete the trial. Nonetheless, in both studies, clients whose animals lost weight reported sequential improvement in activity and quality of life, with a sequential reduction in food-seeking behaviour.

A critical factor in the management of overweight and obesity is caregiver adherence to recommendations. Veterinarians commonly recommend dietary energy restriction with a commercially available, nutritionally balanced, weight loss diet as the primary strategy for weight loss in overweight and obese animals. While these have been successful in experimental settings, they are not as successful in real-world clinical settings [56]. Slow progress was associated with reduced client adherence or discontinuation of caloric restriction. In a study comparing weight loss outcomes between dogs and cats with Class I and Class II obesity, those classified as having Class II obesity lost weight more slowly and lost more lean tissue mass when on controlled weight reduction than their Class I counterparts [15]. Physiological adaptations to weight loss might also increase the challenge of caloric restriction. For example, altered concentration of appetite-related hormones, decreased energy expenditure secondary to reduced body mass and enhanced metabolic efficiency, altered nutrient metabolism and increased perception of hunger may increase animal appetite (and associated behaviours) and increase the rate of weight gain [15]. This may make caregivers feel disheartened and disengaged.

### 3.5. Impact of Overweight and Obesity and Their Management in Companion Animals, Categorised According to the Five Domains Model of Animal Welfare

#### 3.5.1. Domain 1: Nutrition and Hydration

Hunger motivates animals to eat. Voluntary food intake may lead to positive affective states, including masticatory pleasure, the pleasure of salt taste, the pleasure of a variety of food tastes, smells and textures, postprandial satiety (i.e., feeling full and no longer motivated to eat) and gastrointestinal comfort [57]. Prolonged or excessive restricted food intake may lead to general hunger, cravings for salt, or, in extreme cases, weakness of starvation [13]. Caregivers may interpret the Five Freedoms and Provisions model of animal welfare [58] to be absolute so that “dogs and cats should never be hungry” and that hunger indicates poor welfare. In seeking to ensure animals are free from hunger, caregivers may overfeed animals, leading to overweight and eventually obesity. However, Mellor argues that experiences of thirst, hunger and pain are required to drive drinking, eating and avoidance or withdrawal from things that cause injury, so-called “survival critical” behaviours [59], meaning total freedom from hunger is not a normal/natural state. Conversely, overeating can lead to negative affective states such as feeling bloated, overfull, experiencing gastrointestinal pain, nausea or malaise [13].

##### Food Formulation and Selection

Dietary management of overweight and obese animals involves considering both food quantity and quality, which can influence physiological and behavioural responses.

Dogs are facultative carnivores with taste and selection preferences for protein [60], particularly meat [61] and a flexible food range which includes cooked foods [62] and natural sugars (such as fructose, found in fruit) [63]. This, in part, explains why dogs may enjoy foods prepared for human consumption. A diet selection study of laboratory-housed dogs reported that when given the opportunity, dogs self-select a consistent macronutrient ratio of 30% protein: 63% fat: 7% carbohydrate by energy [64]. AFFCO provides minimum (and maximum when toxicity can occur) nutrient content for pet foods to prevent disease [65], such that animals are required to eat the recommended volume to ensure they are attaining adequate nutrition. When developing a weight loss plan based on reducing caloric intake by reducing food volume, the proportionate drop in macro and micronutrients may fall below minimum requirements for some nutrients, including protein [66]. This may trigger hunger, food-seeking behaviours or even hunger-related aggression [67]. It can be difficult to determine the right amount to feed an animal, as stated proportions on retail-bought foods can be misleading, incorrect [68] or take time and education for animal caregivers and veterinarians to interpret [69]. Furthermore, nutritional requirements can vary widely depending on energy use, genetics, environment and life stage [70]. Some weight loss diets may not provide optimal nutrition and may therefore negatively impact the animal’s health and welfare [66,69,71].

Weight management diets which address issues of hunger and satiety by reducing fat content, increasing fibre content and maintaining protein, vitamin and mineral proportions [72] may better meet an animal’s health requirements. A meta-analysis of studies assessing the effects of macronutrients and overall energy intake on weight and body composition of obese dogs to determine optimal nutritional levels for promoting weight loss found that diets with energy densities of less than 3.275 kcal, protein levels over 25%, total dietary fibre over 12%, fat less than 10% and non-nitrogenous extract of less than 40% supported healthy weight loss, reduced body fat and the preservation of lean muscle mass [7]. Furthermore, a high protein diet (48.7%) enriched with omega fatty acids and isoflavones (from soy germ meal) preserved lean muscle mass during weight loss compared to a metabolic weight control diet (26.5% protein) [73].

Animals have dietary preferences. Dogs may preferentially select fatty foods exceeding their caloric requirements [74], suggesting a preference—at least in the short term—for high-fat foods.

Cats, as obligate carnivores, have a narrow, small-mammalian-prey-driven dietary range [48,75]. They also have a metabolic ceiling for metabolising carbohydrate [76] and select a high protein and fat macronutrient ratio (p:f:c) at between 52:46:2 (by metabolisable energy (ME)) [77] and 52:36:12 (by ME) [76]. Cats are insensitive to sugars and relatively insensitive to salt [63]. Diets which deviate from these parameters may induce dysregulation of digestion [71,76], and behaviour changes related to poor satiation, such as increased food seeking and ingestion [63]. Affective states resulting from inappropriate dietary options such as hunger or inadequate satiety may contribute to overeating [78].

One reason that caregivers of overweight and obese dogs resisted weight management plans was concerns that an animal “might suffer” [79]. It is undeniable that restriction of caloric intake can have a negative impact on the welfare of animals. Increased hunger, food-seeking behaviour and anxiety in animals may lead caregivers to abandon weight loss efforts for their animals. In studies evaluating weight loss in client-owned obese dogs and cats, non-adherence was reported by 17/19 dog owners [80] and 9/12 cat owners [81]. Hunger in animals with existing behaviour problems may lead to an escalation in arousal and lower thresholds of reactivity [82] including for behaviours such as guarding or excessive vocalisation, highlighting the need to take into consideration the individual’s mental state when making recommendations to reduce food intake [83].

#### 3.5.2. Domain 2: Physical Environment

The physical environment encompasses the thermal and structural properties of an animal’s environment. Being overweight and obese can lead to thermal discomfort, particularly in warm environments, and is a risk factor for heat stroke [84,85]. Heat stroke can lead to negative affective states such as weakness, dizziness, breathlessness and pain [13]. Confined environments such as crates, kennels, or travelling in cars may exacerbate thermal discomfort and mechanical stress in overweight and obese animals compared to those in ideal body condition [86]. The physical environment also includes surfaces, and overweight or obese animals kept on hard surfaces may experience exacerbation of osteoarthritic pain. Slippery surfaces may present an increased injury risk for these animals, which again may lead to negative affective states such as pain and debility. Environmental interaction constraints may include reduced opportunity for cats to access high resting spots because of weight-related mobility impairment [11,33] and limited access or comfort for dogs in confined spaces such as a kennel or crate.

#### 3.5.3. Domain 3: Health

Overweight and obesity may have metabolic and pathophysiological sequelae and increase the risk of comorbidities. Obesity is associated with adverse clinical consequences of expanded adipose tissue and fat deposition, which lead to metabolic dysregulation, insulin resistance, hormonal derangements and inflammation [16,87,88,89]. These in turn lead to comorbidities, functional impairment, and decreased quality of life, including but not limited to diabetes mellitus (particularly in cats), dysregulation of circulating lipids, pancreatitis, musculoskeletal conditions including osteoarthritis, cardiovascular disease, pulmonary dysfunction, intestinal bacterial dysbiosis, neoplasia, cognitive decline, respiratory disease, dermatological disease, urinary tract disease, urinary incontinence, hepatopathies, changes to renal architecture and increased risk of death due to heat stroke [5,89,90,91,92]. Conversely, excessive caloric restriction may also negatively impact health (e.g., primary hepatic lipidosis in cats [93]).

Increased loading on joints can lead to musculoskeletal discomfort or pain. For example, a case–control study of cats found that owners of overweight and obese cats were twice as likely to report early degenerative joint disease (DJD)-related signs than cats that were not overweight [94]. Obesity was also associated with a higher rate of anaesthetic mortality in dogs [95] and cats [96], though the latter finding was not confirmed in a recent study [97]. On the other hand, overweight and obesity have been correlated with increased survival times in dogs and cats with heart failure, and dogs with renal disease (the so-called “obesity paradox”) [98].

Non-pathologic weight reduction leads to alleviation of adverse physiological and welfare consequences of obesity. In a study of client-owned dogs attending an obesity clinic, weight loss reduced insulin resistance (evidenced by reduced insulin concentrations and insulin:glucose ratio), and subclinical inflammation associated with obesity [99]. Weight loss was associated with reduction in systolic blood pressure, cholesterol, triglyceride and fasting insulin [100], resolution of left ventricular hypertrophy [92], and improvement in subclinical renal function [101]. A study of clinically lame obese dogs with radiographic signs of osteoarthritis found that body weight reduction by 6.1% or more significantly decreased clinical lameness, with kinetic gait analysis supporting improvements with a body weight reduction of 8.85% or more [102]. Weight loss improved oxygenation, but not ventilation, in dogs undergoing sedation [103]. Obese client-owned dogs who completed a weight loss program had significantly increased vitality scores, and decreased scores for emotional disturbance and pain [104]. 

Whether reduced longevity in itself is a welfare issue is contentious, as welfare is seen as a property of a conscious, sentient animal, while death may be considered a welfare-neutral state [105]. However, it has been argued that death is a welfare issue in that it deprives animals of the opportunity to experience future positive welfare states [106]. Furthermore, reduced longevity may reflect reduced health. A higher BCS was negatively correlated with median longevity and reduced median time to onset of chronic disease in a long-term, controlled experiment involving 48 Labrador retrievers [107]. Compared with control dogs, dogs fed 25% less weighed less, had lower body fat content, lower serum triglycerides, triiodothyronine, insulin, and glucose concentrations, and significantly higher median lifespan with delayed onset of clinical signs of chronic disease, including osteoarthritis. There was no significant difference in maximum lifespan between the two groups. In a retrospective case–controlled study of client-owned, middle-aged, neutered dogs attending veterinary hospitals across North America (*n* = 50,787), the risk of instantaneous death was greater in overweight dogs across all ages, while the median life span was up to 2.5 years shorter in overweight compared with normal-weight dogs [108].

The impact of overweight and obesity on longevity appears nuanced in cats. In a study of client-owned cats from a single feline-dominant practice in Sydney, cats with a BCS of <5 or 9 had reduced survival and lifespan [109]. However, those with a BCS of 6–8 had the longest lifespan and survival. Nonetheless, the findings strongly suggested that a BCS of 9 reduces longevity in cats.

#### 3.5.4. Domain 4: Behavioural Interactions

Overweight and obesity and related conditions may constrain behavioural interactions with the environment, other animals (including conspecifics, other household pets and prey) and humans (particularly with caregivers with whom the strongest and most influential attachments occur [110]). Such reduced opportunities for behavioural interactions may negatively impact an animal’s welfare.

##### Interacting with the Environment, Including Appetitive, Consummatory and Species-Typical Behaviours

Animals interact with their environments through species-typical appetitive behaviours. Appetitive behaviours are defined as motivated, goal-seeking behaviours which are temporally separate from, but often precede, consummatory behaviour [111]. Foraging is a form of appetitive behaviour which engages a dog’s or cat’s significant olfactory abilities [112,113,114], along with their other senses, to track and source food. It employs decision-making skills, such as determining what is worth pursuing (e.g., prey or scavenging) [63,115], and the form of behaviour required, including lay-in-wait (a common feline strategy), ambush or chase and pounce (common canine strategies [116]) for prey. These behaviours promote agency and sensory stimulation [117], improve fitness and expend energy. Other examples of appetitive behaviours are searching (following a scent [118], climbing and digging), investigating food items (sniffing and licking [119], pawing and playing [120]), carrying an item to a preferred location, and throwing, bowing and pouncing behaviours [120]. Dogs and cats can remain motivated to perform appetitive behaviours even where these are functionally unnecessary, such as when they are fed their daily ration in meals at regular intervals [121,122]. Contra-freeloading describes when animals choose to work to attain food over freely available food. A dog’s willingness to engage in contra-freeloading increases with BCS [123]. Likewise, kennelled dogs offered a food enrichment toy spent more time performing appetitive behaviours and being active and barking less frequently compared to a control group not provided with food enrichment [124].

Free-ranging cats spend between 40 min and 3 h in hunting behaviours (including lay-in-wait) per rodent captured [125]. When caregivers feed animals immediately, the performance of such appetitive behaviours is truncated [121]. Caregivers may misinterpret an animal engaging in appetitive behaviour (such as waiting by the kitchen close to mealtimes) as requiring food immediately. It is possible that some animals spend more time eating to compensate for the loss of opportunity to express appetitive behaviours [78].

Free-ranging dogs tend to eat 4 to 8 meals per day [63], largely during daylight hours, unless dehydrated or undernourished [71]. Cats extend to nocturnal feeding periods, eating 8–12 small meals throughout the day and night [121,125]. Both species follow diurnal patterns likely to reflect evolutionary factors [125,126,127] and that prey are more active at dawn and dusk. Free-ranging animals spend substantial amounts of time and energy obtaining food, with varying success rates [128]. This unfulfilled motivation may be a frustration for companion dogs and cats kept indoors [11]. Obese dogs spent less time engaged in vigorous activity [129], which is associated with an increased risk of obesity [130].

Activity choices are influenced by age, as young animals may spend more time mouthing and chewing to learn and to play [131], while aged pets are more likely to have dental [132], osteoarthritic [133], or other pain which may affect the type of activity and foods they can comfortably manage. Therefore, it is important to consider age and comorbidities when devising a weight management strategy.

##### Interacting with Other Species and Humans

Scheduled walks provide opportunities for companion dogs to engage in positive behavioural interactions with conspecifics, to sniff or forage to interact with real or artificial prey [134,135] and spend time with their human caregivers. However, less bonded human–dog dyads walked less frequently, and the dogs had higher BCSs than strongly bonded dyads [136].

In domestic conditions, feeding animals is a human behaviour driven by routine and reinforced by positive interactions with animals [137]. For domestic dogs and cats, reduced frequency of feeding [7] or a complete restriction on feeding of treats may be associated with reduced positive interactions with caregivers who feel giving treats strengthens the bond with their pet [138,139]. Feeding may be employed by caregivers to distract or occupy an animal displaying unwanted behaviours such as vocalising. Feeding may be useful to classically counter-condition such behaviour where a negative emotional state is present [140].

More frequent feeding was not linked with increased likelihood of overweight or obesity in dogs and cats [130,141]. Indeed, in cats, less frequent feeding was associated with higher obesity rates [141].

- Learned behaviour

Begging or food-seeking behaviours represent a mechanism through which companion animals can actively participate in the timing, form and quantity of food they eat. These behaviours are a form of negotiation with humans [142], often rewarded by the provision of highly palatable food. The expression of these behaviours varies across individual human–animal dyads, influenced by factors such as personality and expectations of both the animal and the caregiver [142].

**Figure 2:**
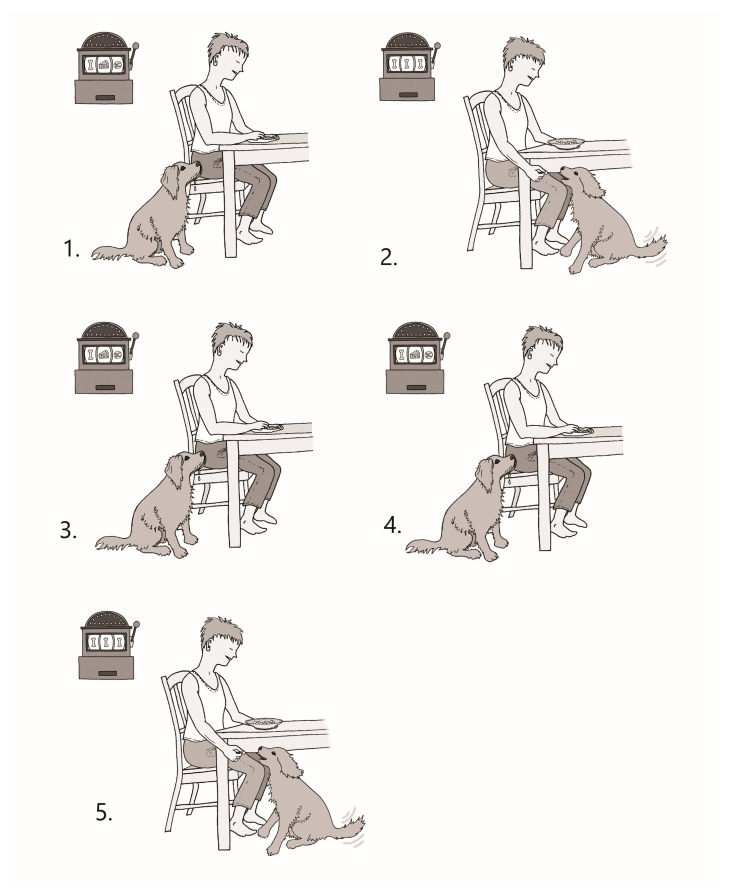
Depiction of how intermittent reinforcement perpetuates behaviour. In this example, the behaviour is food-seeking from the table (**1**). The animal approaches the caregiver and waits for food (like putting money in a poker machine and pulling the lever) (**2**). The caregiver feeds the animal from the table, rewarding this behaviour (like winning on the poker machine) (**3**). The animal enjoys the reward but resumes waiting (like winning on the poker machine, then immediately playing again in the hope of winning again) [148] (**4**). The animal learns to persist until rewarded (playing repeatedly) (**5**). After varying durations (playing on the poker machine), food is offered (like winning on the poker machine). Because there is some chance of ‘winning’, the animal learns to persist, even if receiving food rarely (intermittent reinforcement strengthens the behaviour).

**Figure 3:**
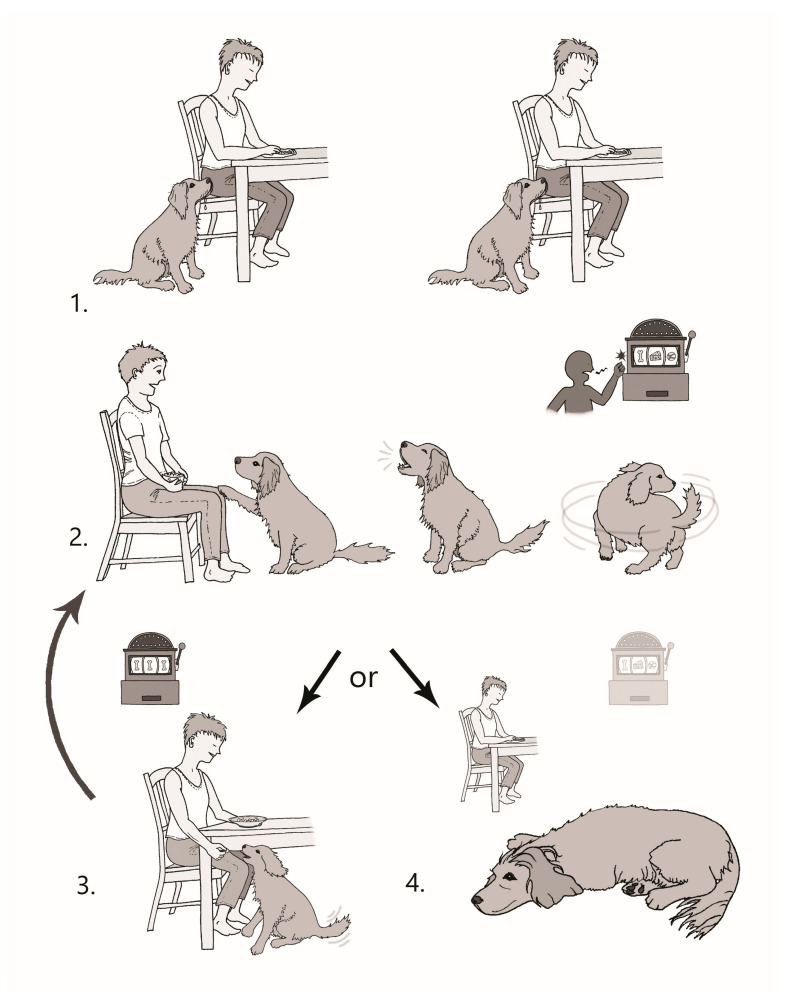
Depiction of an extinction burst. An animal performs food-seeking behaviour (begs) because this behaviour has previously been rewarded (like winning on a poker machine) (**1**). However, the caregiver stops providing food from the table when the animal approaches (this is like playing the poker machine and never winning) (**2**). When food is not given, the animal intensifies their behaviour. This is known as an extinction burst (like banging or shaking the poker machine to force release of the reward). These more intense behaviours can be frustrating and distressing for the animal and the caregiver (**3**). Extinction burst behaviours are very hard to ignore, so food may eventually be offered (like winning on the poker machine). Rewarding this behaviour reinforces it, so the animal learns to perform the more intense behaviour next time and the cycle repeats (curved arrow) (**4**). If extinction burst behaviours are never reinforced, they are eventually extinguished [150] (this is like learning one cannot control the poker machine, so playing stops).

Within animal–caregiver dyads, learning theory and ethology underpin how both parties behave. Behaviour is based on previous experience and interactions, particularly within the dyad [143,144]. Thorndike’s Law of Effect describes operant conditioning, whereby behaviours followed by rewarding outcomes (reinforcement) are more likely to recur [145]. In practice, begging and food-seeking behaviours are hard for caregivers to ignore, and most will often or always give a treat if the animal begs [146]. Periodic treat offerings inadvertently maintain the behaviour through intermittent reinforcement, thereby strengthening an animal’s persistence (see Figure 2) [147]. The animal learns that a reward occurs after a variable number of attempts or time, which increases the likelihood of continued performance of the behaviour, meaning animals learn to persist until they receive desired food [148]. Extinguishing these entrenched behaviours requires the complete absence of reinforcement over an extended period. Withdrawal of reinforcement often triggers a temporary escalation in the behaviour—known as an extinction burst—before the behaviour diminishes [34,148] (see Figure 3). Extinction bursts may be interpreted by caregivers as signs of deprivation or indicative of animal needs, prompting the provision of food. Alternatively, extinction bursts may be considered nuisance behaviour. Caregivers may provide food in the hope that the behaviour will stop. Regardless, this behaviour is stressful for both animals and caregivers. The feeding of animals is a behaviour driven by routine and habit [137] and reinforced by interactions with animals. The reduction of the feeding of treats or table scraps might be rejected if it requires the exclusion of an animal from family life [149].

#### 3.5.5. Domain 5: Mental State

Domains 1–4 focus on factors that give rise to positive or negative affective states that contribute to an animal’s mental state [11]. For example, overweight and obesity may be associated with negative experiences related to poor fitness, such as physical weakness and debility [13] (see Table 1). Furthermore, diet and consummatory behaviours may directly influence both affective states and the effectiveness of management strategies in overweight and obese patients.

Behaviour problems have been shown to decrease when the form and presentation of food items are modified to increase behavioural choices and encourage species-typical behaviours [121,151]. Examples of behaviour problems alleviated using food affordance methods include feline vocalisation [152], canine humping behaviour [153], problem barking [124] and food-seeking [122]. Conversely, restricting food intake for animals who have behaviour problems or that are already stressed may destabilise mood (defined as a non-triggered, usually low-intensity emotional state [154]). This may contribute to hunger-related food guarding [155,156,157] or deterioration of behaviour [155].

While appetite and food intake primarily serve to meet nutritional and energy requirements, they also engage a range of affective states as components of consummatory behaviour. Consummatory behaviours—innate, reflexive actions involved in food ingestion—depend on adequate motor function and motivation to perform them [111]. For example, dogs typically consume their daily ration within five minutes [158], yet factors such as flavour enjoyment, oral satisfaction, and orogastric comfort can significantly influence the quantity consumed, the degree of postprandial satisfaction, and, ultimately, the animal’s affective states. In dogs, slowing down the rate of ingestion allows for activation of the parasympathetic nervous system [159], the component of the autonomic nervous system important for homeostasis, including digestion. Conversely, rapid eating and reduced masticatory effort stimulate the opposing sympathetic nervous system (SNS) [159].

Diet, behaviour and affective states are interwoven in complex bi-directional systems via neurohormones and endocrine hormones such as cortisol, serotonin, endorphins, dopamine and noradrenaline. Serotonin is one of the primary neurohormones involved in food ingestion and mood. Serotonin receptor antagonism, leading to a deficiency in the central serotonergic system, is associated with increased food consumption and weight gain [160], while serotonergic stimulation is associated with reduced food consumption, satiety, and positive mood [160]. In a study of healthy client-owned dogs recruited from a Korean veterinary teaching hospital, lean dogs had higher peripheral serotonin concentrations than obese dogs [161]. Fat consumption stimulates dopaminergic and endorphin pathways and plays a role in reward systems and satiety [162]. Polyunsaturated fatty acid (omega-3 and omega-6) concentrations also play a role in improving behaviour, health and mood [163,164]. These are considerations when reducing dietary fat intake.

Arousal stimulates the sympathetic nervous system, mediated by cortisol and noradrenaline, and can be associated with loss of appetite, for example, when animals experience separation-associated distress when left alone. A causal association in dogs was reported by Turnbull et al. (2025) between an increase in body fat and an increase in hair cortisol concentrations, an indicator of the stress response [165]. Conversely, animals may eat to cope with negative emotional states, known as stress-induced or emotional eating [78]. Caregivers reported emotional eating more in dogs with medical conditions, behaviour problems or that were considered unhappy [158]. In dogs, chewing provides a putative behavioural strategy to reduce stress, and the ill health and welfare sequelae which chronic stress may induce [166,167]. Dogs that are stressed may seek chewing opportunities not for the calories but to gain the arousal-mediating benefits of the behaviour [167,168].

In summary, overweight, obesity and their management impact animal welfare and behaviour. Importantly, management of overweight and obesity may lead to (unintended) negative as well as (intended) positive affective states. This is important to understand and consider when developing strategies to address overweight and obesity, as these can impact animal behaviour, animal–human interactions, and caregiver adherence.

### 3.6. Barriers to Veterinary Management of Overweight and Obesity in Dogs and Cats

It has been argued that veterinarians are to some extent “abdicating their role as advocates for animals” by failing to adequately address the companion animal obesity epidemic, given the known negative impacts on animal welfare [169,170]. Proponents of this perspective argue that veterinarians should consistently:

- Pre-emptively monitor animals to prevent obesity from developing;
- Inform clients when their animal is overweight;
- Inform clients about the health and welfare implications of obesity;
- Provide information on how to achieve weight loss; and
- Provide information and strategies on how to manage behaviour needs other than feeding [169].

A key challenge is caregiver recognition of overweight and obesity. Some caregivers may not be aware that obesity is a health concern. In a survey of dog and cat owners in the US and Australia in 2006, 32% reported living with an overweight or obese animal, while 0.3% perceived obesity to be a health concern [171]. However, in a 2023 survey of Swedish dog owners, 99% agreed that being overweight may lead to negative consequences [172]. It may be that awareness of the negative impacts of overweight and obesity has increased over time.

There is some evidence that animal caregivers exhibit “weight blindness”, that is, a tendency to underassess the BCS of normal-weight animals and a failure to identify overweight animals. This was shown in the Swedish study cited above, where groups of owners assessed BCS in dogs both from photographs (with 75% of respondents underestimating BCS) and on their own dog (with 35% of owners underestimating their own dog’s BCS compared to assessment by a veterinary team member) [172]. In a study where dogs and cats were presented to a veterinary teaching hospital in France over a three-year period, 27% of dog owners and 24% of cat owners underestimated their animal’s BCS [173]. Where overweight and obesity are prevalent in dogs, a dog in ideal body condition appeared relatively thin to their owners. However, so-called weight-blindness can be addressed by veterinary team members: while dog owners initially significantly differed from veterinarians in their assessment of their dog’s BCS, once told verbally how to conduct body condition scoring, given a practical demonstration, and provided with the WSAVA 9-point Body Condition Scale and printed directions on how to use it, their assessments were as accurate as those of veterinary team members [172].

Veterinary clients may not attribute the welfare impacts of overweight and obesity to their animal’s body condition. For example, a survey of dog owners incorporating a health-related quality of life (HRQOL) assessment tool found that overweight and obese dogs had significantly reduced HRQOL compared with those with a healthy body weight [174], particularly in the domains of energetic/enthusiastic and active/comfortable [175]. Overweight and obese dogs did have a higher median score in calm/relaxed than non-overweight dogs, which could reflect misinterpretation of reduced activity by owners. In another survey of dog owners, HRQOL was reduced in overweight and obese dogs compared with dogs with a healthy body weight [175]. It is also possible that owners may dismiss signs related to obesity as “old age”. In a study comparing cross-sectional surveys of veterinary professionals and dog owners, one of the main reasons for owners not taking their dogs to the vet was a belief that clinical signs (including sleeping all the time, slowing down on walks and being stiff on rising) were normal signs of ageing [176]. These could be signs of musculoskeletal disease, which can be exacerbated by overweight or obesity.

But it is possible that veterinarians understate or even avoid communicating findings of overweight and obesity if they feel this will alienate the client, jeopardising future interactions and thus the potential to positively influence the animal’s welfare [177]. Other reported reasons for not raising the issue of animal overweight and obesity with a client included time constraints, a client’s history of poor adherence, concern about causing offence, the client themselves being obese, lack of communication skills, lack of knowledge about overweight and obesity and lack of knowledge about nutrition [178,179]. Veterinarians did not consistently report a finding of overweight and obesity to clients [25,50]. When they did, it was often simply to warn clients against further weight gain [149] or to tell people to reduce caloric intake and increase output [50]. Most veterinarians did not communicate specific recommendations (e.g., what to feed, how much to feed, how to overcome potential barriers to calorie restriction, how to increase physical activity). Where they did, clients did not always remember. For example, in interviews of 74 dog owners and 24 veterinarians across four first opinion practices in the UK, 92% of veterinarians said they provided advice on achieving weight loss, yet 15% of owners could not recall specific advice given about weight loss, and only 20 percent reported having a clear management plan [178]. The authors concluded there was scope for improvement in the identification and communication of overweight and obesity assessment and weight loss advice.

One potential reason for suboptimal communication and recommendations may be inadequate dietary history, making it difficult to provide tailored recommendations [149,180]. In an analysis of 98 video-recorded consultations containing a discussion of nutrition drawn from companion animal practices in Ontario, Canada, veterinary dietary histories focused predominantly on the commercial diets fed to animals [180]. Clients mentioned human foods and treats at low frequencies, despite the feeding of such foods being shown to be more frequent in previous studies. For example, in a cross-sectional survey of dog and cat owners (*n* = 3673) in Australia, Canada, New Zealand, the UK and USA, most animals were fed treats, snacks and table scraps or leftovers in addition to their main diet [181].

In observations of consultations where cats were identified as overweight or obese, clients resisted weight management recommendations due to the challenge of caloric restriction in the context of feeding multiple animals in a household, commitment to treat-giving, concerns about food palatability, and difficulty changing from free-feeding to feeding measured amounts. When presented with these barriers, veterinarians tended to partially or completely abandon their recommendations, and/or drop the topic altogether [149]. Veterinarians may refrain from making recommendations if they do not believe that their recommendations will make an impact.

Studies suggest that many animal caregivers are not ready for change, according to the States of Change transtheoretical model, which conceptualises five stages to describe how individuals move through the process of intentional change. For example, an online questionnaire of dog and cat owners who self-reported their animals as overweight or obese (*n* = 119) found that 52.1% were in the precontemplation stage, and 42% were in the contemplation stage, where readiness to change is low [182]. Those in the precontemplation stage had little awareness of reasons for change and no commitment to changing, while those in the contemplation stage might have been considering addressing their animal’s weight but were not yet committed to action. Few respondents were in the action or maintenance stages. The authors noted that when clients are ambivalent or undecided about change, they may respond to arguments for change with apparent counterarguments. However, this apparent “resistance” may reflect an early phase of the change process. Provision of unsolicited advice or a plan during the precontemplation phase without client involvement might increase resistance. In this early stage, provision of accurate information about weight, body condition score and the impacts of overweight and obesity on health and welfare, combined with exploration of the client’s own perceptions of their animals’ weight, may be more beneficial.

A factor that may negatively impact weight management is time constraints in veterinary settings. While there are an increasing number of resources available that support veterinarians in discussing overweight and obesity management with clients, it is impossible to deliver such protocols adequately within the time allocated for a typical veterinary consultation [50].

The current American Animal Hospital Association (AAHA) recommendation is to undertake nutritional screening on every patient. This involves:

1. Assessing BCS/Muscle Condition Score (MCS);
2. Weighing the patient;
3. Assessing change in body weight since the previous visit/over time;
4. Assessing dietary history;
5. Assessing activity level;
6. Assessing the animal’s at-home environment; and
7. Performing a comprehensive physical examination.

Non-ideal BCS/MCS, significant weight gain or loss, change in activity levels, inadequacy or inappropriateness of physical environment, abnormalities on physical examination, abnormalities in diagnostic tests, a poorly balanced diet, a treat allowance constituting >10% of dietary intake, or indicators of altered gastrointestinal function (vomiting, diarrhoea, nausea, flatulence, constipation) should trigger an extended evaluation [183].

For weight management, such an evaluation would include:

1. Determination of the current caloric intake;
2. Calculation of the daily caloric requirements for weight loss (see https://24051120.fs1.hubspotusercontent-na1.net/hubfs/24051120/Guidelines%20PDFs/Nutrition%20and%20Weight%20Management/nutritiongl_box1.pdf (accessed on 8 April 2026);
3. Calculation of a safe rate of weight loss for this animal (typically 1–2% body weight per week);
4. Making precise dietary recommendations (including treats);
5. Preparing detailed meal and exercise plans;
6. Scheduling regular follow-up appointments;
7. Adjusting the plan as needed;
8. Reverting to a weight maintenance plan when goals are achieved.

The reality is that this is very demanding in a clinical setting. The number of veterinarians who fully engaged with this process was low, and—disappointingly for those who put in the effort—success was not correlated with the level of engagement [50]. High-level veterinary engagement (VE) (Level 3—see Table 2) peaked with dogs at BCS 7. Veterinary engagement at any level increased overall with dogs at BCS 8 and 9, while VE3 declined. This suggests that veterinarians engage heavily with weight loss programs only when the problem is advanced and is consequently harder to manage.

The current recommendations focus on both detailed, accurate history taking regarding diet and lifestyle, with no explicit consideration of animal welfare and behaviour considerations, nor barriers to human behaviour change. Furthermore, there is an assumption that a complete history will be provided. Caregivers control companion animal access to calories, either directly through feeding animals or indirectly through permitting scavenging. They control the quality and quantity of food available, as well as the extent to which an animal exercises, and may not disclose the quality and quantity of the diet if they feel such disclosure would lead to negative judgment [50].

While veterinarians tended to discuss the type of food recommended for weight management, specific recommendations regarding the volume of food to feed were not consistently provided. A cross-sectional survey of dog and cat owners (*n* = 1402) in Canada and the USA found that only 34.2% of respondents relied on their veterinarian’s recommendations on what volume of food to feed [184].

As discussed in Section 3.5, one factor rarely acknowledged in the literature as a barrier to weight loss in animals is caregiver concerns regarding the negative welfare impacts of management of overweight and obesity in dogs and cats. It is not known whether frank acknowledgement of welfare trade-offs [185] (for example, the negative welfare impacts of overweight and obesity vs. the negative welfare impacts of caloric restriction) would yield improved client adherence to veterinary weight management plans.

Similarly, weight management based on a commercial diet may not be acceptable to some animals accustomed to eating a non-commercial diet, or caregivers who do not wish to feed their animals a commercial diet. If an animal only eats a particular form of food (dry, semi-dry, wet, raw), it may be difficult to transition them to a different form of food. An international survey of dog and cat owners revealed that the feeding of non-commercial and “unconventional” foods increased between 2008 and 2018 [181]. The authors speculated that this change may be due to loss of caregiver trust in the pet food industry due to product contamination and mass recalls, increased humanisation of companion animals and lower confidence in veterinarians with respect to nutrition.

Another barrier to management of overweight and obesity is caregiver reluctance to “force” animals to exercise lest this cause suffering, discomfort and pain, for example, through exacerbation of musculoskeletal conditions. Recommendations for increased exercise may not be followed due to time constraints, lack of client motivation and, in the case of dogs, patient factors including fear and reactivity, which can prohibit exercise in shared public spaces. Caregiver concerns regarding veterinary clinic-associated fear, anxiety and stress behaviours of dogs and cats may act as a barrier to adherence to attend monitoring of body weight and BCS visits. Recommended management strategies influence the mental state of animals. Ideally, weight management plans require weight and body condition score to be assessed and recorded every 2–4 weeks [6]. If this monitoring is done in a veterinary setting, it may lead to fear, anxiety and stress among some patients, negatively impacting welfare [186,187].

There is also a possibility that veterinarians and caregivers prioritise different outcomes. While veterinarians tend to stress impacts of overweight and obesity on QOL, there is some evidence that caregivers may be more motivated by the impact on longevity. A survey of dog owners primarily from Canada found that negative impact on life expectancy was the most important factor that would encourage them to pursue weight management in an overweight or obese dog (relative importance 29%) followed by timeline for development of arthritis (19%), future QOL (19%), change to cost of food (19%) and future mobility (14%) [188]. Similarly, a survey of cat owners primarily from the US and Canada found that negative impact on life expectancy was the most important factor that would encourage them to pursue weight management in an overweight or obese cat (relative importance 33%) compared with cost of food (20%), future quality of life (20%), future mobility (14%) and risk of developing diabetes (12%) [189].

Another potential barrier to communication around overweight and obesity between veterinary team members and veterinary clients is implicit weight bias. Weight stigma (negative stereotypes and prejudices regarding weight) and weight stigma by association (for example, stigma towards parents of overweight children) have been documented in human healthcare settings [190]. Weight stigma in human healthcare is associated with poor communication with patients, manifesting as negative attitudes and stereotypes, insensitive and stigmatising language, attribution of causes of obesity to individual choices, less patient-centred approaches, less rapport and discussions, banal weight loss advice and assumptions that all patient symptoms are due to weight [191]. In turn, patients feel disrespected, judged or blamed, have lower trust in healthcare providers, receive reduced quality of care, have poorer treatment adherence, increased clinical attrition, and may delay or avoid care altogether [191]. In a cross-sectional survey of Canadian veterinarians and veterinary technicians incorporating an implicit association test (IAT), participants rated owners of overweight dogs as less effective caregivers than owners of lean dogs [190]. Intriguingly, participants rated owners of overweight cats as more caring than owners of lean cats. The authors speculate that having a lean dog may suggest that the dog is having regular exercise, and thus being well looked after, whereas having a lean cat may be considered to reflect a less close relationship [190]. The IAT results showed that 90% of participants demonstrated some degree of unconscious preference for lean clients over overweight clients. As this study was a survey based on hypothetical scenarios, it is not known how this bias might impact communication between veterinary team members and animal caregivers in reality.

Outside of clinical settings, information regarding overweight and obesity in dogs and cats may not be accessible or useful. A review of the readability of websites containing information on pet obesity found that most were inaccessible due to high reading grade levels, with less than 25% providing information on body condition scoring [192].

#### Alternative Strategies for Weight Loss Management: Medication and Genetics

Recent developments in human medicine in the management of diabetes have yielded medications with potential application for weight loss in animals. These include glucagon-like peptide 1 (GLP-1) receptor agonists, GLP-1/Glucose-dependent insulinotropic polypeptide (GIP) agonists, GLP-1/GIP/Glucagon agonists, GIP antagonist/GLP-1 agonists and GLP-1/amylin agonists [193]. Some weight loss drugs can be administered orally, or as long-acting subdermal implants, which—when available for veterinary use—may increase client adherence when medicating cats in particular [194].

In 2007, dirlotapide, a microsomal triglyceride transfer protein inhibitor (marketed as Slentrol), entered the market in Europe and the US. It held great promise, especially for those animals who had failed to lose weight with dietary restriction and exercise. In one study, treated dogs lost 19.3% weight after 12 weeks (with no dietary restriction), regaining only 3.4% after the drug was discontinued. Early weight loss in the trial led to increased activity in the dogs, which led to further weight loss. Yet the drug was subsequently withdrawn in Europe by the European Medicines Agency due to side effects including emesis, diarrhoea, lethargy and anorexia. It was later withdrawn in the US by the manufacturer due to lack of interest from caregivers—in part because of the negative impact of the dog’s reduced appetite on the human–animal bond [195]. Medication that suppresses appetite may challenge caregivers and veterinarians alike as it can be difficult to distinguish appetite reduction as the desired drug effect from appetite reduction due to concurrent illness. Medication may play a role in weight loss of animals but is unlikely to be acceptable to clients if they feel that weight loss is at the expense of their animal’s welfare or the human–animal bond.

Now that genetic testing is available, and genetic risk factors for high food motivation have been identified, the possibility of eliminating these factors via selective breeding has been raised [196]. However, as with medication, there may be unintended consequences, including reduced trainability and impacts on the human–animal bond. Currently, there is not enough evidence to support breeding to eliminate genes associated with food motivation.

## 4. Discussion

Overweight and obesity are common among dogs and cats. While it is true that overweight and obesity stem from an excess of energy intake relative to consumption, the literature on obesity suggests that it involves the derangement of complex homeostatic mechanisms controlling energy balance, and it is simplistic and unhelpful to regard it as a moral failing of animals and their caregivers [89]. Furthermore, caregivers may be concerned about negative welfare and behaviour implications of managing overweight and obesity via caloric restriction and increased physical activity. We have shown that animal welfare and behaviour considerations are central to understanding the impact of overweight and obesity. As this review demonstrates, despite the prevalence and known health and welfare risks of overweight and obesity in dogs and cats, there are animal-, caregiver- and veterinarian-related barriers preventing effective identification and management. We suggest that some of these are related to a narrow focus on overweight and obesity as disorders of nutrition, without considering animal welfare and behaviour. Reducing dietary energy intake using a purpose-formulated weight loss diet without attending to behaviour is unlikely to succeed and may compromise the welfare of animals and the wellbeing of their caregivers.

The focus of this discussion will be on practical steps that primary care veterinarians might take to prevent and manage overweight and obesity in their patients.

### 4.1. Assess, Record and Share Weight and Body Condition Score at Every Consultation

To ensure that overweight and obesity are identified, every animal seen by a veterinarian should be weighed and a BCS recorded [37,170]. Prior to sharing this information with clients, it may be helpful to ask what they think about their animal’s BCS and weight. This helps inform the veterinary team of the next best steps—whether to invest time justifying their assessment that the animal is overweight, providing information about overweight and obesity, or developing and sharing a management plan [137]. Regardless, both body weight and BCS should be shared with clients at each visit [20]. Additionally, we recommend that techniques for body condition scoring be demonstrated to clients, and all clients should be given the appropriate resources so that they can undertake body condition scoring at home [197,198], or be able to access information about how to perform body condition scoring online in a readable format [192]. Body weight and BCS should be measured at every consult, including in puppies and kittens, as overweight and obesity can occur in young animals [26], and may be easier to address early in life. Additionally, incorporating muscle condition scoring in examinations of animals from middle age onwards may help support the health and welfare of older animals by triggering earlier detection and management [27] of sarcopenia and sarcopenic obesity [5].

Where practical, regular weighing of animals is recommended as food intake can be adjusted to prevent weight gain or loss. Animals deemed to be overweight or obese, and those in weight loss programs, should be weighed weekly to fortnightly. For patients fearful of veterinary clinics, caregivers can be encouraged to purchase scales to weigh animals at home. A baseline weight should be taken on home scales, as there can be a discrepancy between these and clinic scales. As electronic scales become more affordable, there is scope for the use of passive “smart” scales incorporated in food dispensers or litter boxes, or placed beneath beds, to weigh cats and small dogs without the need for handling [52]. Telemedicine may facilitate body condition scoring by veterinary team members in collaboration with the client, as well as troubleshooting of weight management strategies, particularly for cats [199]. If a fearful animal must visit the hospital, pre-visit pharmaceuticals may be suitable. Ideally, avoid separating the patient from the client [200,201].

### 4.2. Allow Time for an Extended Evaluation and Follow-Up of Patients Deemed Overweight or Obese

As most cases of overweight and obesity are diagnosed incidentally in dogs and cats, it is important to address these concerns as they arise. Visiting the veterinarian is inconvenient and involves a certain level of stress for clients and animals. One of the barriers to accessible veterinary care is a lack of transport [202,203]. However, the diagnosis of overweight or obesity should trigger an extended evaluation [183]. Therefore, some flexibility in the schedule is required to allow veterinary team members to assess the animal’s weight, gather appropriate information and develop a plan, rather than require the client to return to address a problem they were not aware of and may not be convinced of the importance of addressing. Following the physical examination, there may be scope for some of this extended evaluation to occur asynchronously, for example, via telemedicine.

### 4.3. Take a Complete and Accurate Dietary History

Dietary recommendations are likely to be better accepted if they are based on a complete and accurate dietary history [180]. As part of its Nutrition and Weight Management Guidelines [183], the American Animal Hospital Association (AAHA) has developed a list of open and closed-ended questions for veterinarians that may help elicit a comprehensive dietary history [183]. It is important to include questions regarding meals as well as treats, training rewards and foods in which medication is administered. In our experience, it is important to ask the client not just what the animal is intentionally fed, but also any items not intentionally fed but eaten. For example, animals in multi-animal households may eat the leftovers of other animals. Similarly, animals living in households with infants may eat food that has been dropped or discarded. To ensure a comprehensive dietary history, it may be helpful to provide the client with a pre-appointment questionnaire. This information can also be collected by veterinary nurses, technicians or trained veterinary support staff, freeing up clinician time.

### 4.4. Take a Complete and Accurate History Relating to Behaviours Associated with Eating, Activity and Lifestyle

While the literature on management of overweight and obesity focuses on taking a comprehensive dietary history, it is just as important to consider behaviour and physical activity which may help prevent overweight and obesity in mature animals and preserve lean muscle mass in senior animals [27]. This should include feeding schedule and routine, behaviour in relation to seeking and eating food, food preferences, interactions with other animals, physical activity including opportunities for play, and potential challenges anticipated by the caregiver.

This information can be useful in identifying specific barriers and opportunities for weight management. Satiety and mood enhancement may occur through activities other than food consumption, such as positive interactions with humans or other animals in the household or beyond, play, exercise and being in natural light [204,205]. Making caregivers aware of these strategies may promote adherence. Recommendations should consider animal preferences, behaviour and lifestyle. For dogs, physical exercise does not necessarily mean lead walking. Exercise within the animal’s home and yard may be more successful, particularly with dogs that cannot be socialised with other dogs. For cats, helping owners recognise and understand their cat’s need for play or other enrichment is important. Veterinarians can suggest suitable activities and toys [206].

### 4.5. Provide Tailored Dietary Recommendations Including “What” and “How Much” to Feed

Dietary recommendations should be tailored to the patient and caregiver. Recommendation of a diet based on a similar form of food may be more acceptable and better promote adherence. There is scope for veterinarians to provide information about the specific volume of food [183]. This is challenging in animals fed a diet consisting of different types of foods (for example, those fed an entirely home-prepared diet, those fed a combination of commercial diets or those fed a mix of commercial diets and home-prepared diets). The Association for Pet Obesity Prevention provides resources, including a resting and maintenance energy requirement calculator and daily calorie counters for dogs and cats for animal caregivers (https://www.petobesityprevention.org/veterinary-resources accessed on 8 April 2026) which may aid in the formulation of specific advice. An alternate approach involves reducing the total calories fed, which may involve switching to lower-calorie diets and reducing portion sizes. The use of a smaller-sized bowl may reduce stress in caregivers who feel they are otherwise giving their animal a small portion [6].

### 4.6. Allow for Treat-Feeding in Dietary Plans

Allowances should be made for animals that are habituated to receiving treats or require food rewards to shape behaviour or to facilitate medicating animals. Treats and food rewards provide an opportunity for positive human–animal interactions and may be critical for the human–animal bond [207]. Giving caregivers low-calorie treat options, such as vegetables, allows them to continue rewarding animals while avoiding excessive caloric intake [6]. Alternatively, setting aside a portion of the daily ration to be dispensed as rewards for appropriate behaviour, and allocating 10% of daily calorie intake as treats [208] allows caregivers to continue to train and positively interact with animals without increasing total daily caloric intake [207]. As these contribute to the total caloric intake [183], medication-associated treats should be factored into the daily ration.

### 4.7. Recommend Strategies to Increase Appetitive and Consummatory Behaviours, and Slow the Rate of Ingestion

Engagement in appetitive and consummatory behaviours promotes species-typical activities (e.g., foraging, chewing, play, sniffing) that require minimal human input and may support weight maintenance or loss. These changes may assist animal caregivers in feeling that they are adding something to an animal’s life, rather than depriving them [138].

Species-typical behaviour opportunities for dogs include ‘sniffaris’ to engage with outdoor scents [113], cardboard boxes to tear up, digging pits, bubbles, tunnels and interactive equipment [209] and nose work (finding and interacting with hidden and novel scents). Examples for cats include a toy on a tether (such as Kong™ active twisted boa), a ruler under the bed covers (requiring some human input), a high perch at a window to observe the outdoors and climbing opportunities such as sisal-covered poles or cat towers. Although these are food-free activities, in some cases they may be paired with the administration of a treat to truncate the search and catch sequence [120]. The provision of long, edible grass (in pots indoors, or in an enclosed outdoor area), and sensory gardens [210] are options for both species.

**Figure 4:**
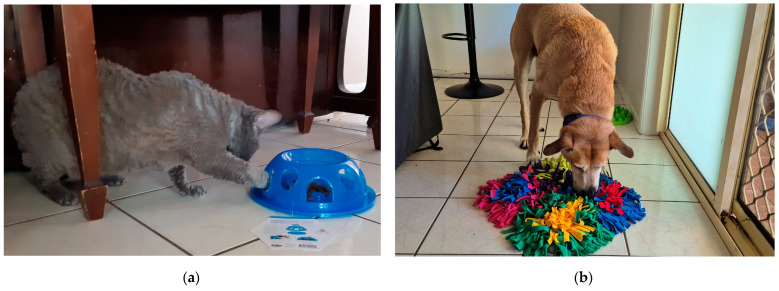
Strategies designed to increase appetitive behaviours and slow down the rate of ingestion of food. (**a**) depicts a cat engaging with a puzzle feeder; (**b**) depicts a dog eating their dry food ration from a snuffle mat. Images taken by Dr Rimini Quinn.

Other strategies involve presenting the animal’s usual diet in ways which slow ingestion while adding interest and effort. Cats can be provided with treat dispensers (Figure 4a), puzzle feeders and opportunities to find food placed around the house [120]. Water may be added to dry food to increase the bulk to slow the rate of eating and improve satiety [122,211]. Offering stuffable items such as Kong™ toys (Colorado, USA), treasure hunts (hide small pieces of food around a room) or a snuffle mat (Figure 4b) provides energy-consuming activities likely to be engaged in by overweight and obese pets.

Veterinarians can help clients overcome barriers by suggesting time-efficient techniques such as scatter feeding, separation of animals prior to feeding in multi-animal households and cost-effective modes of presenting food, such as using cardboard boxes and recycled containers. It is prudent to ensure an animal’s capabilities match the foraging task to prevent unfulfilled effort and frustration [212], which may lead to disengagement and negative affective states.

In some cases it is possible to slow the rate of ingestion by altering the type of food offered, though health considerations should be taken into account and potential risks discussed with clients beforehand [213,214,215,216]. For example, offering commercial chews or whole bones, which take time to gradually dismember, provides more opportunity for consummatory behaviours than food items which can be rapidly consumed, such as minced meat or commercial food.

### 4.8. Recommend Strategies to Reduce Food-Seeking Behaviour

The provision of engaging activities may reduce food and attention-seeking around mealtimes. Potential benefits are positive distraction and increased energy use. In our experience, commonly reported barriers to offering such activities include time constraints, a belief that the animals will not show interest, challenges in multi-animal households (such as resource-guarding) and costs. However, these approaches have been shown to be effective in dogs and cats. In a survey of dog owners, daily enrichment feeding was associated with decreased begging and increased satiety [122]. In a four-week-long, randomised controlled trial of 92 client-owned companion cats, where one group was provided food puzzles and one group was provided with non-food-related toys, the prevalence of attention-seeking behaviour decreased significantly, regardless of whether enrichment involved object-play or food puzzles [217]. This finding suggests that object-play or food puzzles were a viable solution to attention-seeking behaviours in cats.

**Figure 5:**
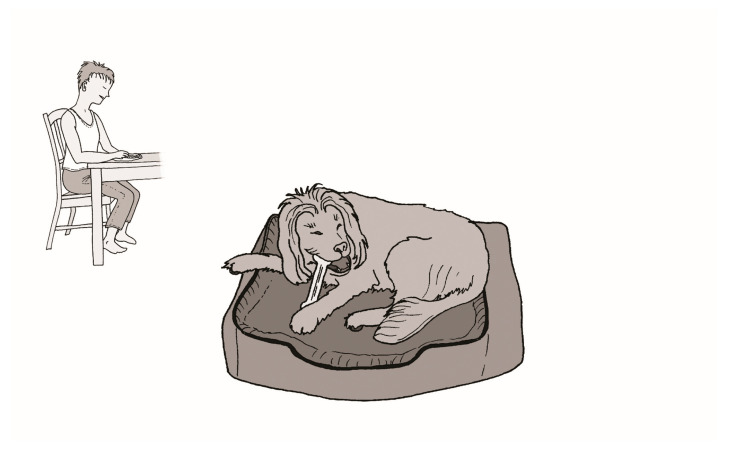
An alternative, welfare-friendly approach to prevent or remediate food-seeking behaviour using behaviour modification involves pre-emptively providing an alternative activity, such as giving a chew or lick, on a mat or bed. Alternatives need to be sufficiently valuable so that the animal is motivated to engage with them.

We believe that it is critical that caregivers are informed about extinction bursts (for example, in animals used to being fed from the table) so they can anticipate and prepare for these. Rather than attempting to ignore food-seeking and begging behaviours, including extinction burst behaviour, which can prove very difficult for caregivers, we recommend avoiding situations that trigger the approach behaviour altogether. This involves providing a positive, incompatible alternative (i.e., behaviour that the animal cannot perform at the same time as unwanted behaviour). For example, the caregiver can provide an animal with a chew or lick option on their mat or bed prior to sitting down at the table to eat (Figure 5). Note that the offering needs to be of high enough value so that the animal is motivated to engage with this option rather than approach the caregiver at the table. To extinguish the behaviour, it is critical that no food is offered from the table by any person at any time (including visitors) [150].

### 4.9. Recommend Feeding Smaller Meals More Frequently than Fewer, Larger Meals

The digestion of food itself requires energy (thermogenesis [218]). Where total daily energy requirements are supplied in several small meals, there is more energy consumed than if the total daily requirement were fed in a single meal (David Fraser, personal communication, 23 January 2026).

Feeding dogs smaller meals twice daily and diurnally, and giving cats access to small, regular meals, including during the night, may aid in improved satisfaction and reduce frustration. Automatic food dispensers may help with metered nocturnal food access. At least part of the daily ration can be fed using food-toys or puzzle feeders [211], which can help distribute caloric intake across time.

### 4.10. Recommend Feeding Strategies for Multiple-Pet Households

Multiple-pet households provide a challenge to caregiver adherence, as animals on a calorie-restricted diet may eat their ration as well as that of other animals. Additionally, the threat of competition for food may increase the rate of ingestion. Veterinarians can recommend practical strategies, including relocation of feeding stations (for example, cat bowls can be moved onto benches or shelves where dogs cannot reach), separation of animals during feeding, and use of microchip feeders, which can help ensure that animals access only their ration and reduce overconsumption [52,122,219]. In our experience, it is helpful to suggest that where animals are separated, bowls, food remnants and feeders should be removed before they are reunited to avoid social tension between animals and animals “cleaning up” after each other.

### 4.11. Explain the Benefits of Weight Loss to Caregivers

The benefits of weight loss may be obvious to veterinary team members, but may not be clear to caregivers, who may see no benefit to the animal or themselves in altering their behaviour [46]. Benefits may include improved welfare (evidenced perhaps by improved engagement with humans and other animals, increased activity and play), improved health (including alleviation of symptoms of comorbidities like osteoarthritis) and increased longevity.

### 4.12. Manage Caregiver Expectations

Caregivers should be informed that weight loss is gradual, results may not be evident in the short term and that additional consulting time is required for the development of a tailored weight management plan and follow-ups. It may be helpful to openly acknowledge trade-offs, including increased hunger and food-seeking behaviour, in animals on weight management programs. When discussing welfare compromise associated with overweight and obesity, the Five Domains model may be helpful as it emphasises affective states rather than absolute freedom from hunger.

A realistic approach to weight loss might be setting the target weight as higher than the ideal weight, to improve animal function and quality of life, and build client confidence, while minimising the risk of premature discontinuation of caloric restriction [15,182].

## 5. Future Directions

While there is a sizeable amount of literature on overweight and obesity in companion animals, there remains a need for evidence-based, effective interventions for overweight and obese companion dogs and cats living in diverse real-world settings. While there are important reasons for highly controlled trials of dog and cat diets in experimental settings to eliminate confounders and standardise observations, their success in real-world settings can be influenced by contextual factors including animal factors (such as genotype, breed, sex, neuter status and age), human factors (including lifestyle, social determinants of health, food selection and feeding routines, number of animals in the household), human–animal interactions (including interactions and the human–animal bond) and environmental factors (including obesogenic environments) [47,220]. Most published studies of human–dog interactions, including how humans care for dogs, are limited to Western, Educated, Industrialised, Rich, Democratic (WEIRD) societies [221]. However, there is marked variation in human–dog interactions and relationships that impact dog care outside of these contexts, which may not be reflected in the current literature [221]. Expansion of studies on the prevalence of and risk factors for overweight and obesity in non-WEIRD contexts is required as a foundation for contextually appropriate management strategies.

There is a need to develop standardised, evidence-based guidelines for optimal macronutrient levels for effective weight loss that support cell renewal [7] at different life stages for both dogs and cats. There has been an emphasis on the development of commercial weight loss diets that maximise satiety [56]; however, a greater emphasis on presenting foods that promote and consider species-typical behaviours and the broader role of satiety, appetitive and consummatory behaviour is warranted. The development of weight loss and maintenance diets that focus not simply on meeting minimum nutritional needs, but also on the macronutrient ratio, form and presentation of dietary offerings to meet animal preferences may improve caregiver adherence to weight management plans.

While much of the literature discussed is focused on identifying and addressing barriers to weight loss in dogs and cats, studies of caregivers of formerly overweight and obese animals who have successfully lost weight (and maintained weight loss) through adherence to weight loss plans may provide insights into effective strategies.

Given concerns about client adherence to weight management plans, it would be useful to evaluate the impact of electronic or take-home, tailored information to clients about their animals’ BCS, weight, and weight management. The development and trial of a health pack designed to help owners manage the weight of dogs found that around 80% of caregivers changed the way they fed, exercised or interacted with their dog during an eight-week trial period, with 92% attributing these changes to the health pack [222]. Further research including larger trials and the development and trial of a similar resource for cat caregivers is required to determine the efficacy of these resources in supporting caregivers in managing the weight of their animals. Similarly, as noted by Cairns-Haylor and Fordyce, the impact of providing different forms of continued client support outside of the initial consultation could be further investigated [178]. The success of weight loss plans is typically defined as the percentage of body weight lost, reaching a target weight or achieving a particular rate of weight loss. However, given the aim of improving welfare, it may be preferable to focus primarily on welfare-based outcomes such as quality of life assessment, decreased severity of comorbidities and mobility [53].

Artificial intelligence (AI) is increasingly used as a tool for animal welfare assessment, particularly in the context of farm animals [223,224]. Using ethical guardrails [225], there is scope to explore generative AI in the assessment and monitoring of veterinary patients. Another possibility would be to develop and validate an AI instrument designed to analyse detailed responses to dietary questionnaires provided by caregivers (including information about animal preferences, behaviours and routines) to develop a tailored, staged weight loss and maintenance plan for each patient which can be rapidly updated in the light of response.

A systematic review of human behaviour change interventions targeting weight, body condition and body fat of dogs found that most had a very small or null effect on a dog’s weight and body fat [226]. There remains a need for well-designed, controlled studies that are sufficiently powered to detect potentially minor effects of weight loss interventions in dogs and cats, as these may help refine weight management plans. Further studies are required to determine whether even a modest loss of weight may aid in sustaining caregiver motivation to adhere to weight loss plans.

Finally, from both the “One Health” [227] and “One Welfare” [228] perspectives, where animal, human and ecological health and welfare are seen as interconnected and interdependent, there is a need to explore broader strategies to address obesogenic environmental factors, including reduced opportunities for exercise and behavioural interactions, for the benefit of animals and humans.

## 6. Conclusions

Overweight and obesity are major problems for companion dogs and cats, with potential negative impacts across all five domains of animal welfare. Available evidence suggests that veterinary interventions have low success rates. We argue that a complete understanding of overweight and obesity needs to consider behaviour and animal welfare concerns. Management requires understanding and empathy regarding real-world challenges faced by caregivers. Good veterinarian–client communication is essential in assessing and managing overweight and obesity. This requires sufficient time to adequately research, evaluate, design, record and monitor weight management plans. The ultimate goal of managing overweight and obesity is improving the animal’s welfare by achieving both a healthy BCS and promoting positive affective states. Further research is required to determine successful weight loss strategies for overweight and obese dogs and cats.

## Figures and Tables

**Table 1:** Negative experiences that may be associated with overweight and obesity ^1^.

Experience	Description
Physical discomfort	Joint stiffness due to cramped space, and/or the unpleasantness of being on hard or rough surfaces
Thermal discomfort (too hot)	Distress of overheating/inability to cool down or stay cool
Exhaustion	A negative feeling associated with physical and/or mental fatigue linked to excessive metabolic demands (e.g., physical activity)
Weakness	A negative feeling associated with reduced strength, muscle tone, vigour, or fitness
Breathlessness	An urgent compulsion to increase respiratory activity (e.g., breathing rate and depth; gasping), to overcome resistance to airflow due to obstructions in air passages (e.g., laboured breathing), or to escape from external impediments to breathing; can lead to anxiety, fear, and panic
Debility	A negative feeling associated with physical weakness, especially associated with illness

^1^ Adapted from [13].

**Table 2:** Veterinarian engagement scores, associated descriptions and engagement with clients from a retrospective review of electronic medical records for dogs (*n* = 500) assessed as overweight or obese ^1^.

Level of Veterinarian Engagement (VE)	Description of Engagement	Percentage of Veterinarians
0	Diagnosis only. No documented discussion with client in medical record.	5.7%
1	Basic recommendation of “… eat less, exercise more”.	48.7%
2	- Determination of percent and amount overweight - +/−ideal body weight calculated - Recommendation of percentage caloric reduction - Increase activity	31.3%
3	Recommendation of 1 and 2 above plus: - Calculation of current caloric intake, kcal/day to feed - +/−actual measurement of feed - +/−recommended weight rechecks - +/−RX diet recommendation - +/−written instructions	14.3%

^1^ Adapted from [50].

## Data Availability

Not applicable.

## Abbreviations

The following abbreviations are used in this manuscript:

AI	Artificial intelligence
ANZCVS	Australian and New Zealand College of Veterinary Scientists
AWSEL	Animal Welfare Science, Ethics and Law
BCS	Body condition score
DEXA	Dual-energy X-ray absorptiometry
DORA	Dog Obesity Risk and Appetite questionnaire
ECAWBM	European College of Animal Welfare and Behaviour Medicine
MCS	Muscle condition score
POMC	Pro-opiomelanocortin
